# Prevalence and distribution of most common ICU pathogens in a Thai-university hospital during a 5-year period

**DOI:** 10.1186/1753-6561-5-S6-P244

**Published:** 2011-06-29

**Authors:** S Boonsong, P Chongtrakool, S Srisangkaew, P Santanirand

**Affiliations:** 1 Department of pathology, Ramathibodi Hospital, Mahidol University, Bangkok, Thailand

## Introduction / objectives

Nosocomial infection remains a major problem among ICU patients which may lead to lethal outcome. Data analysis of prevalence and antibiotic resistance profile of bacterial isolates from ICU could help improvement of treatment, control and prevention of infection.

## Methods

Microbiological data during year 2006-2010 of Ramathibodi Hospital, a 900-bed Thai-University hospital in Bangkok, Thailand, were retrospective analysed. All bacterial isolates were identified by conventional biochemical tests as recommended in Manual of Clinical Microbiology. *Candida* species were identified according to characteristics of chlamydoconidia production and carbohydrate assimilation/fermentation profile using 13/6 carbon substrates.

## Results

From year 2006 to 2010, the main nosocomial pathogens in the ICU were *Candida albicans* (16.9%, 17%, 18.2%, 19.4% and 19.5% respectively), followed by non-fermentative gram negative bacilli either *P. aeruginosa* (11.3%, 12%, 12.2%, 15.3%, 14.3% respectively) or *A. baumannii* (12%, 11.3%, 14.7%, 13.1%, 17.7% respectively) or *Staphylococcus* coagulase-positive (10.7%, 11.75%, 9.45%, 10.3%, 7.6%). The second most common gram-negative bateria were either *Escherichia coli* (8.1%, 8.5%, 6.4%, 6.8%, 8.0%) or *Klebsiella pneumoniae* (7.5%, 6.9%, 7.9%, 6.6%, 8%) which approximately half of them were ESBL producers.

## Conclusion

In last 5-year period, the prevalence of nosocomial infection by *C. albicans* and *A. baumannii* in our ICU is rising, while the prevalence of ESBL-producing bacilli is quite stable. The same incidents were reported by many hospitals worldwide. This may be due to the change in preference of antimicrobial agents used by clinician which need further analysis.

## Disclosure of interest

None declared.

